# Do plant populations on distinct inselbergs talk to each other? A case study of genetic connectivity of a bromeliad species in an Ocbil landscape

**DOI:** 10.1002/ece3.3038

**Published:** 2017-05-23

**Authors:** Karina Vanessa Hmeljevski, Alison Gonçalves Nazareno, Marcelo Leandro Bueno, Maurício Sedrez dos Reis, Rafaela Campostrini Forzza

**Affiliations:** ^1^ Rio de Janeiro Botanical GardenRio de JaneiroRJBrazil; ^2^ Department of BotanyUniversity of São PauloSão PauloSPBrazil; ^3^ Department of BotanyLaboratory of Ecology and Evolution of PlantsUniversity of ViçosaViçosaMGBrazil; ^4^ Federal University of Santa CatarinaFlorianópolisSCBrazil

**Keywords:** Atlantic Forest, conservation genetics, *Encholirium horridum*, gene flow, landscape genetics, phylogeography, species distribution models

## Abstract

Here, we explore the historical and contemporaneous patterns of connectivity among *Encholirium horridum* populations located on granitic inselbergs in an Ocbil landscape within the Brazilian Atlantic Forest, using both nuclear and chloroplast microsatellite markers. Beyond to assess the *E. horridum* population genetic structure, we built species distribution models across four periods (current conditions, mid‐Holocene, Last Glacial Maximum [LGM], and Last Interglacial) and inferred putative dispersal corridors using a least‐cost path analysis to elucidate biogeographic patterns. Overall, high and significant genetic divergence was estimated among populations for both nuclear and plastid DNA (Φ_ST__(*n*)_ = 0.463 and Φ_ST__(plastid)_ = 0.961, respectively, *p *< .001). For nuclear genome, almost total absence of genetic admixture among populations and very low migration rates were evident, corroborating with the very low estimates of immigration and emigration rates observed among *E. horridum* populations. Based on the cpDNA results, putative dispersal routes in Sugar Loaf Land across cycles of climatic fluctuations in the Quaternary period revealed that the populations’ connectivity changed little during those events. Genetic analyses highlighted the low genetic connectivity and long‐term persistence of populations, and the founder effect and genetic drift seemed to have been very important processes that shaped the current diversity and genetic structure observed in both genomes. The genetic singularity of each population clearly shows the need for in situ conservation of all of them.

## 1 INTRODUCTION

Dispersal is notoriously recognized in the literature as a key process in determining the spatial population structure of species (Nathan & Muller‐Landau, 2000). Therefore, knowledge of historical dispersal and the resulting gene population flow is crucial to understanding the population biology and evolution of species in their habitats (Howes et al., 2009; Lowe & Allendorf, 2010; Manel & Holderegger, 2013; Yu et al., 2015). The elucidation of biogeographic patterns through the conciliation of palaeoclimatic modeling methods with genetic analysis is a growing area of interest in landscape genetics (Chan, Brown, & Yoder, 2011; Collevatti et al., 2012; Lowe & Allendorf, 2010; Manel & Holderegger, 2013; Melo, Lima‐Ribeiro, Terribile, & Collevatti, 2016; Storfer, Murphy, Spear, Holderegger, & Waits, 2010; Vitorino, Lima‐Ribeiro, Terribile, & Collevatti, 2016), which is a field whose conceptual basis is founded in landscape ecology, population genetics, and spatial statistics (Manel & Holderegger, 2013; Manel, Schwartz, Luikart, & Taberlet, 2003; Sork & Waits, 2010; Storfer et al., 2007). For instance, Collevatti et al. (2012) showed that phylogeographical analyses coupled with species distribution modeling allowed a more powerful framework for evaluating alternative hypotheses and disentangling the mechanisms involved in the origin of the disjunct distribution for a neotropical seasonally dry forest plant species.

For distinct Brazilian biomes, this approach has been explored in order to indicate potential long‐term climate stability areas (refugia) during Quaternary climatic fluctuations (Bueno et al., 2016; Carnaval, Hickerson, Haddad, Rodrigues, & Moritz, 2009; Carnaval & Moritz, 2008; Dantas et al., 2015; Martins, 2011; Werneck, Costa, Colli, Prado, & Sites, 2011; Werneck, Nogueira, Colli, Sites, & Costa, 2012). Despite gaps in the knowledge about palaeoenvironments in Brazil, it is known that complex climatic variations during the Quaternary shaped a large portion of the current landscapes in the country (Behling, 2002; Neto, Galvani, & Vieira, 2015; Oliveira et al., 2005). The interplay between Pleistocene climatic oscillations and Pliocene/Miocene orogenic events seems to have contributed to shaping the current diversity and distribution of modern lineages in South America (Turchetto‐Zolet, Pinheiro, Salgueiro, & Palma‐Silva, 2013).

In coastal Atlantic Forest of eastern Brazil, there is a particular region named Sugar Loaf Land (de Paula, Forzza, Neri, Bueno, & Porembski, 2016), where a humid tropical climate combined with occasional tectonic activity in the past has created a “sea of hills” landscape (Salgado, Bueno, Diniz, & Marent, 2015). This area is inserted basically in the Araçuaí orogen (Alkmim, 2015), which has an internal zone dominated by high‐grade gneiss and granitoid inselbergs that formed between 625 and 490 Ma (Gradim et al., 2014; Varajão & Alkmim, 2015). The exposure of these features could have started sometime around the beginning of the Neogene (c. 20–2.6 Ma), and more recent tectonic motions might have contributed to their final sculpture (Varajão & Alkmim, 2015).

Inselbergs are a special class of residual landforms with a landscape configuration featuring a contrast of prominent elevations (height > 100 m) with surrounding plains (Lima & Corrêa‐Gomes, 2015). They occur isolated or in groups, separated by a few or many kilometers, forming inselberg landscapes. Ecologically, they are characterized by a range of harsh conditions (Lüttge, 2008), such as a high degree of insolation, high evaporation rates, very restricted local soil occurrence, and low water availability (Porembski, 2007; Szarzynski, 2000). These edaphic‐climatic characteristics promote the occurrence of specialized vegetation comprising a great number of endemic species (Porembski, 2007). Specifically, Sugar Loaf Land comprises a specialized Bromeliaceae flora, influenced by climatic factors and regional species pools, and seems to have played a pivotal role in the speciation of many rupicolous members (de Paula et al., 2016). The isolated nature of these terrestrial islands is one of the characteristics that make them interesting models for studies of the genetic connectivity of populations (Porembski, 2007; Porembski & Barthlott, 2000). Strong genetic structure has been repeatedly verified for species in this environment, which indicates an association between low gene flow and high isolation among populations (Barbará, Martinelli, Fay, Mayo, & Lexer, 2007; Boisselier‐Dubayle, Leblois, Samadi, Lambourdière, & Sarthou, 2010; Byrne & Hopper, 2008; Millar, Coates, & Byrne, 2013; Palma‐Silva et al., 2011; Pinheiro et al., 2011, 2014; Tapper et al., 2014a,b).

A few years ago Hopper (2009) proposed the Ocbil theory, a series of hypotheses that attempt to explain the evolution and ecology of biota on very old, climatically buffered, infertile landscapes (Ocbils), in contrast to Yodfels (i.e., young, often disturbed, fertile landscapes). The author focused on the Southwest Australian Floristic Region, the Greater Cape Floristic Region of South Africa, and the Pantepui region of the Guyana Shield in South America as three of the most significant areas on Earth with Ocbils. However, this theory was recently reviewed and new likely Ocbil regions were pointed out by authors, including inselberg systems, in terrains where Ocbils are interspersed among Yodfels (Hopper, Silveira, & Fiedler, 2016).

In this context, *Encholirium* (Pitcairnioideae, Bromeliaceae) is a Brazilian genus whose distribution is intimately related to rocky outcrops of the Caatinga, Cerrado, and Atlantic Forest domains of Brazil (Forzza, 2005). Although the most recent phylogenies of Bromeliaceae have shown the paraphyly of *Encholirium* (Givnish et al., 2011; Krapp, Pinnangé, Benko‐Iseppon, Leme, & Weising, 2014; Schütz, Krapp, Wagner, & Weising, 2016) and suggested its circumscription within *Dyckia* (Schütz et al., 2016), currently 31 species are still accepted (Forzza & Leme, 2015). Within the genus, *Encholirium horridum* L.B.Sm. is an endemic species of granitic inselbergs of Sugar Loaf Land (Forzza, 2005; de Paula et al., 2016), which makes it a good target species for evaluating the history of genetic connectivity among populations in this Ocbil landscape.

This study used eight species‐specific nuclear microsatellite markers (nSSR, simple sequence repeat) and five chloroplast microsatellite markers (cpSSR) to investigate the genetic diversity and structure of *E. horridum* populations in an Ocbil landscape. The specific aims were to estimate levels of genetic variation and population differentiation, identify spatial population structure and dispersal barriers across populations, and elucidate historical and contemporaneous patterns of connectivity among *E. horridum* populations.

## 2 MATERIAL AND METHODS

### 2.1 Habitat and study species

*Encholirium horridum* occurs on inselbergs located from southern Bahia State to the northern Rio de Janeiro State, mainly in the states of Espírito Santo and Minas Gerais, in southeastern Brazil (Hmeljevski, Reis, & Forzza, 2015). The climate of the area of occurrence can be basically classified as Tropical wet‐dry (Aw; Köppen‐Geiger system), with an average temperature and annual precipitation of c. 24°C and 1,100 mm, respectively (pt.climate-data.org). The climate is characterized by distinct seasons, with most of the precipitation occurring in “summer” while the driest months are in “winter,” which is when the species flowers.

This bromeliad species has abundant populations with thousands of individuals. A study on fine‐scale spatial structure (SGS) and paternity analysis in one *E. horridum* population revealed weak SGS and restricted gene flow with pollination events that did not go beyond 45.5 m (Hmeljevski et al., 2015). Besides this, clonal growth was found to be a rare event, indicating that the species is monocarpic (Hmeljevski et al., 2015). This plant species has a cryptic self‐incompatibility system and is pollinated by vertebrates (Hmeljevski, Wolowski, Forzza & Freitas 2017). Although abundant populations are observed for this bromeliad, it has been categorized as Endangered in the *The Red Book of Brazilian Flora* (Forzza et al., 2013). This is mainly because of the degradation of inselbergs from mining granite, as Espírito Santo State is one of the main Brazilian centers of mining and processing ornamental rocks (Menezes & Sampaio, 2012). Other threats include predation of rosettes and immature fruits by goats and cattle and the invasion of exotic grasses that results in frequent fires (Hmeljevski et al., 2015).

### 2.2 Sampling and DNA extraction

**Figure 1:**
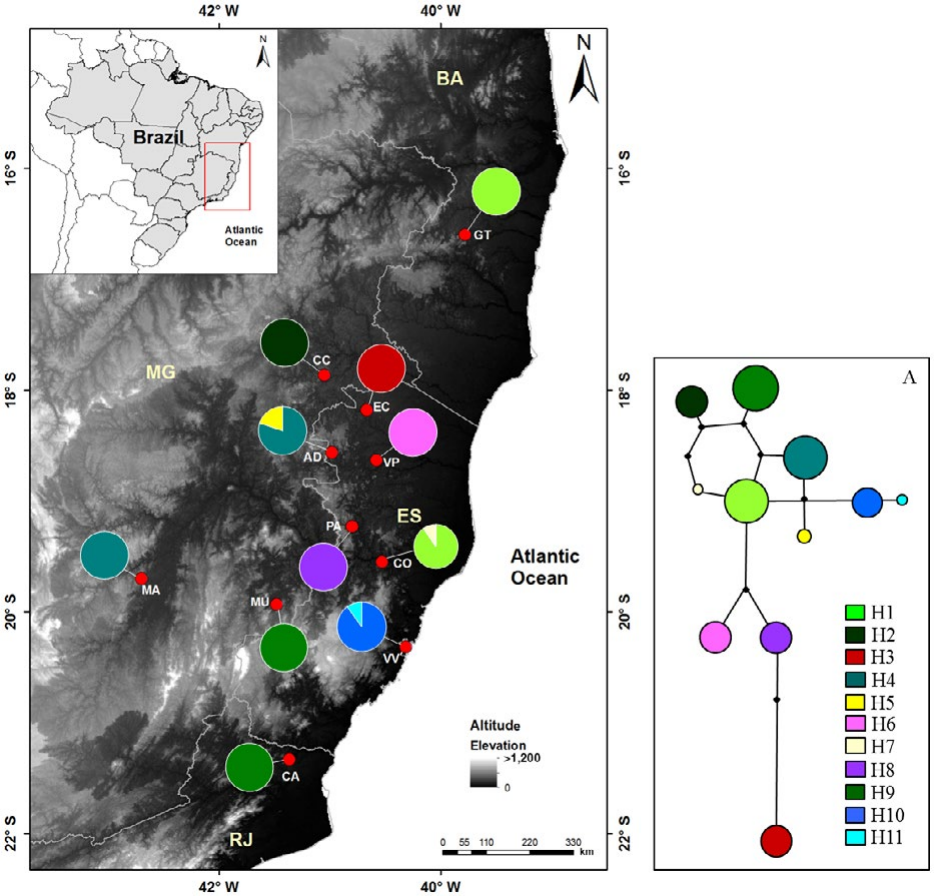
Geographic distribution of *Encholirium horridum* L.B.Sm. sampled populations and respective plastid network (A). Pie charts reflect the frequency of occurrence of each haplotype in each population. Haplotype colors correspond to those shown in networks. In the statistical *Median‐Joining* network, the haplotype frequencies are proportional to circle size. The number of mutations required to explain transitions among haplotypes is indicated along the lines connecting the haplotypes by cross hatches. Small black circles correspond to unsampled or extinct intermediate haplotypes

We collected leaf samples from 526 reproductive individuals of 11 natural populations covering the entire geographic range of *E. horridum* (Figure 1, Table 1). On average, c. 48 and 20 individuals were sampled for nuclear (nDNA) and plastid (cpDNA) genome characterization, respectively (Table 2). Due to the relief's slope, the sampling strategy was to collect from the largest area within populations where it was possible to walk. Weak SGS has been reported for this species (Hmeljevski et al., 2015) so individuals from all *E. horridum* populations were sampled at intervals of at least 10 m to avoid potential sampling of relatives. Leaf material was stored over silica gel at −20°C until DNA extraction. DNA extraction was performed using a NucleoSpin Plant II (Macherey‐Nagel, Düren, Germany) extraction kit.

**Table 1:** Sampling locations in Sugar Loaf Land, Brazil, including population name, geographic region (City and State), latitude and longitude coordinates, and voucher information for *Encholirium horridum* L.B.Sm

Population	City	State	Lat S	Long W	Voucher
GT	Guaratinga	BA	16°36′	39°47′	Daneu 377 (CEPEC)
CC	Carlos Chagas	MG	17°52′	41°03′	Forzza 5775 (RB)
EC	Ecoporanga	ES	18°11′	40°40′	Forzza 5788 (RB)
AD	Água Doce do Norte	ES	18°34′	40°59′	Forzza 5811 (RB)
VP	Vila Pavão	ES	18°38′	40°35′	Forzza 5794 (RB)
PA	Pancas	ES	19°14′	40°48′	Forzza 5838 (RB)
CO	Colatina	ES	19°33′	40°32′	a
MA	Marliéria	MG	19°42′	42°42′	Martinelli 15163 (RB)
MU	Mutum	MG	19°56′	41°29′	Forzza 6057 (RB)
VV	Vila Velha	ES	20°19′	40°19′	Forzza 6083 (RB)
CA	Campos	RJ	21°20′	41°22′	Forzza 6086 (RBvb)

States: BA, Bahia; MG, Minas Gerais; ES, Espírito Santo; RJ, Rio de Janeiro. Herbarium collections: CEPEC, Cocoa Research Center/BA; RB, Rio de Janeiro Botanical Garden/RJ; RBvb, Rio de Janeiro Botanical Garden Bromeliarium.

a There was no fertile individual at the time of sampling.

**Table 2:** Genetic diversity of the 11 *Encholirium horridum* L.B.Sm. populations sampled in Sugar Loaf Land, Brazil

Pop	*n*	Nuclear microsatellites							Chloroplast microsatellites		
		*A*	*R*	*A*_P_	*A*_R_	*H*_*e*_	*F*_IS_	*F*_IS_^1^	*n*	Haplotypes	*h*
GT	53	20	2.38	4	5	0.259	0.429*	0.139	20	H1	0.000
CC	50	72	8.41	11	34	0.668	0.106	−0.029	20	H2	0.000
EC	50	15	1.79	0	3	0.169	0.365*	0.229	20	H3	0.000
AD	47	75	8.97	10	36	0.747	0.239*	0.018	20	H4, H5	0.337
VP	50	95	11.05	17	52	0.833	0.248*	0.092*	20	H6	0.000
PA	50	64	7.55	11	30	0.696	0.150*	0.074*	21	H1, H7	0.181
CO	53	27	3.25	0	6	0.429	0.226*	0.095	20	H8	0.000
MA	35	12	1.50	1	1	0.192	0.221	−0.282	20	H4	0.000
MU	50	64	7.69	15	27	0.725	0.123	0.025	20	H9	0.000
VV	50	34	4.02	1	14	0.373	0.163	0.002	20	H10, H11	0.189
CA	38	10	1.24	1	2	0.010	−0.009	–	20	H9	0.000
Average	48	44	5.26	6	19	0.464	0.206	0.036	20		0.064

See Table 1 for the names of the populations.

*n*, sampling size; *A*, number of alleles; R, allelic richness by rarefaction based on the minimum sample size of 35 individuals; *A*_P_, number of private alleles; *A*_R_, number of rare alleles (frequency <0.05); *H*_*e*_, expected heterozygosity (genetic diversity); *F*_IS_, inbreeding coefficient; *F*_IS_^1^, inbreeding coefficient excluding loci with presence of null alleles; *h*, haplotypic diversity. H1–H11 corresponds to a different haplotype.

Significant values are followed by *.

### 2.3 Simple sequence repeat (SSR) markers and genotyping assays

Eight nuclear microsatellite (nSSR) loci developed for *E. horridum* (EhA07, EhB09, EhC03, EhE01, EhE02, EhE11, EhG03, EhG07; Hmeljevski, Ciampi, Baldauf, Reis, & Forzza, 2013) were genotyped. For cpDNA, the transferability of plastid microsatellites (cpSSR) isolated from *Vriesea gigantea* Mart. ex Schult. & Schult. f. (Palma‐Silva et al., 2009) and *Pitcairnia* spp. (Palma‐Silva et al., 2011), as well as universal primers from *Nicotiana tabacum* L. (Weising & Gardner, 1999), were tested. Eighteen markers were tested, and five polymorphic loci were selected (VgCP2 and VgCP4 from *V. gigantea*; ccmp2, ccmp3, and ccmp6 from *N. tabacum*). Fluorescent‐labeled primers were used for genotyping reactions, following protocols described by Hmeljevski et al. (2013). Both nSSR and cpSSR alleles were resolved in an ABI 3500xL automatic sequencer (Applied Biosystems, Foster City, CA, USA) using GSLIZ600 as the size standard (Applied Biosystems). Locus amplification was performed individually, and the fragments were subsequently pool‐plexed for allele sizing. Allele scoring was made in GeneMapper Software 4.0 (Applied Biosystems).

### 2.4 Prior to genetic analysis

As initial analyses, gametic disequilibrium and null allele presence were tested before including the microsatellite set in the study. Loci that are included in analyses despite gross violations of these assumptions or high rates of error could lead to inaccurate and biased genetic estimates (Selkoe and Toonen, 2006). For nDNA data, linkage disequilibrium was tested for each *E. horridum* population with the program FSTAT (Goudet, 1995). In order to avoid false positives, the *p*‐values (=.05) obtained were adjusted applying a sequential Bonferroni correction for multiple comparisons (Rice, 1989). The presence of null alleles was verified using the software Micro‐Checker 2.2.3 (van Oosterhout et al.*,* 2004).

### 2.5 Genetic diversity analysis

For the nSSR data, genetic diversity parameters were estimated by the number of alleles (*A*), the allelic richness (*R*), the number of private alleles (*A*_P_), the number of rare alleles (*A*_R_, alleles with frequency < 0.05), and expected heterozygosity (*H*_*e*_) in Hardy–Weinberg equilibrium (HWE). Analyses were run using the program FSTAT (Goudet, 1995), except for the private alleles that were estimated in GeneAlEx6.5 (Peakall & Smouse, 2006, 2012), and allelic richness that were calculated in the R package, diveRsity (Keenan, McGinnity, Cross, Crozier, & Prodohl, 2013). In order to make the allelic richness independent from sample size, the rarefaction method was used to standardize it to the smallest sample size. The inbreeding coefficient (*F*_IS_) was estimated considering the presence and absence of null alleles, and its significance (determined by 10,000 permutations across loci) was tested using the program SPAGeDi 1.5 (Hardy & Vekemans, 2002).

For cpDNA data, alleles at cpSSR were combined into haplotypes for each individual. From this, we characterized each population by the number of haplotypes detected and haplotypic diversity (*h*), calculated with the software CONTRIB (Petit, El Mousadik, & Pons, 1998). A median‐joining haplotype network (Bandelt, Forster, & Röhl, 1999) was constructed based on cpSSR genetic variants using NETWORK 4.6.1.1 (http://www.fluxus-engineering.com).

### 2.6 Genetic structure and phylogeographic signal

For both nuclear and plastid DNA, Φ‐statistics (Weir & Cockerham, 1984) were estimated by analysis of molecular variance (AMOVA) to assess patterns of differentiation. This was performed with the software ARLEQUIN 3.5.2.1 (Excoffier & Lischer, 2010) using 10,000 permutations.

Phylogeographical signal was also investigated for both classes of genetic markers by comparing measures of differentiation based on ordered versus unordered alleles (Hardy, Charbonnel, Fréville, & Heuertz, 2003; Pons and Petit, 1996). This reveals the importance of mutation relative to other causes of genetic differentiation (i.e., gene flow and divergence time) (Hardy et al., 2003). For nSSR, the global *F*_ST_ and *R*_ST_ values were compared to examine whether mutations were a significant cause of population differentiation using the *R*_ST_ permutation procedure described by Hardy et al. (2003). Populations that have been isolated for a significant period of time would have accumulated mutations that contribute to their differentiation, and if mutation at the microsatellite loci has contributed significantly to this differentiation, the observed value of *R*_ST_ should be higher than the permuted *R*_ST_ (*pR*_ST_). Conversely, if populations were isolated for a relatively short period of time (in comparison with the mutation rate) or if gene flow has been important, *R*_ST_ would not be significantly different from *F*_ST_ (Hardy et al., 2003). The test was implemented in the program SPAGeDi using 10,000 permutations across allele size. For cpSSRs, the global *F*_ST_ and *N*_ST_ indices of among‐population differentiation were computed in the program SPAGeDI to test whether *N*_ST_ **> *F*_ST_. For the estimation of *N*_ST_, the distance between haplotypes was calculated as the sum of their absolute length differences across all loci. We performed 10,000 permutations of rows and columns of the distance matrix between haplotypes. Such a significant relationship suggests that a phylogeographic signal occurred when distinct haplotypes found in the same population were related more closely (i.e., separated by fewer mutational events) on average than distinct haplotypes sampled in different populations (Duminil et al., 2010).

For the nSSRs and cpSSRs, we estimated the pairwise genetic distance and verified the isolation by distance among populations using SPAGeDi, and Mantel's test with 30,000 randomizations was performed using the program IBDWS 3.23 (Jensen, Bohonak, & Kelley, 2005). Bayesian clustering was also performed, in STRUCTURE 2.3.4, to assign individuals to genetic clusters (*K*) and to estimate admixture proportions (*Q*) for each individual (Pritchard, Stephens, & Donnelly, 2000). The analysis was performed under the assumption that the allele frequencies in different populations can be correlated with one another and that alleles carried at a particular locus by a particular individual originated in some unknown population (admixture model). Analyses were carried out with a burn‐in period of 20,000 and run lengths of 200,000, and 10 iterations per *K*, ranging from 1 to 12, to confirm stabilization of the summary statistics (Pritchard et al., 2000). We first ran the dataset without considering sampling localities, followed by analysis considering this information. In order to estimate the appropriate number of populations, Δ*K* was estimated as an ad hoc quantity related to the second‐order rate of change of the log probability of data with respect to the number of clusters, as proposed by Evanno et al. (2005). The most likely *K* number graphics and barplot figures were generated with the clumpak server on the web (Kopelman, Mayzel, Jakobsson, Rosenberg, & Mayrose, 2015; http://clumpak.tau.ac.il/).

### 2.7 Quantification of migration among populations

For the nDNA data, short‐ (the past one to three generations) and long‐term gene flow (average over the past *n* generations, where *n *= the number of generations the populations have been at equilibrium) was estimated to examine the connectivity patterns of the population. A Bayesian approach was used to determine recent migration rates (*m*), which was implemented in BAYESASS 1.3 (Wilson & Rannala, 2003). Samples were run for 2.0 × 10^7^ interactions with a burn‐in period of 10^7^generations and sampled every 2,000 interactions. To estimate long‐term gene flow, we used the program MIGRATE 3.2.6 (Beerli & Felsenstein, 1999, 2001). This program estimates historical migration rates (*M*) as well as the effective population sizes using Markov chain Monte Carlo techniques and coalescence theory. In this analysis, we used a Brownian motion model of mutation under the maximum‐likelihood framework. We used five independent replicates of 10 short chains 50,000 iterations in length, three long chains 500,000 iterations in length, and four heated static chains at temperatures 1.0, 1.5, 3.0, and 10,000 to increase the efficiency of the searches. The long‐term estimate of gene flow (*M*) was converted to the proportion of migrants (*m*) from population *i* to population *j*, using the formula *m*_*ij*_ = *M*_*ij*_μ (where μ = 10^−3^mutation per allele per generation; Udupa & Baum, 2001), so that these values would be more comparable to estimates of *m* produced by bayesass. Furthermore, we calculated Spearman's ρ coefficient in order to determine whether there is a correlation between short‐ and long‐term estimates of gene flow across population pairs.

### 2.8 Species distribution modeling

*Encholirium horridum* species distribution models (SDMs) were built for current conditions, mid‐Holocene (6,000 years ago‐6 kyr BP), LGM (21,000 years ago‐21 kyr BP), and Last Interglacial (LIG, 120,000 years ago‐120 kyr BP) periods.

Environmental data (19 standard BIOCLIM variables) were obtained for all geographical coordinates, at 1 km spatial resolution, from the WorldClim database (Hijmans, Cameron, Parra, Jones, & Jarvis, 2005), and cropped to the range of Brazil. To avoid over‐parameterization of SDM due to redundant variables (Dormann et al., 2013), the correlations between bioclimatic variables were assessed and those with presumed reduced biological relevance (*r* > .9) were eliminated, which left 14 for subsequent analysis (Table S1). Species occurrence localities totaled 55 points and were mainly based on the collection at the Rio de Janeiro Botanical Garden (RB, http://www.jbrj.gov.br/jabot) and field surveys. SDMs were modeled using Maxent v.3.3 (Phillips & Dudik, 2008). Palaeoclimatic data represent downscaled climate data from simulations with Global Climate Models (GCMs) based on the Coupled Model Intercomparison Project Phase 5 (CMIP5; Taylor, Stouffer, & Meehl, 2012). For the LIG model, the Otto‐Bliesner et al. (2006) approach was used, and for LGM and Holocene, the Community Climate System Model—CCSM4 was employed (Gent et al., 2011). All geographic information system (GIS) analyses were performed in ArcGIS v.10 (ESRI, 2011).

### 2.9 Visualizing dispersal corridors

To understand how environmental conditions and genetic data contributed to shaping the distribution of *E. horridum* in Sugar Loaf Land across the time periods, we inferred corridors of highest dispersal probability, as described in Chan et al. (2011). According to Neto et al. (2015), during the beginning of the last Quaternary glaciation (LIG, 115,000–70,000 years ago), Brazil experienced a period of maximum cold and aridity that had strong repercussions on the vegetation. Climatic fluctuations were frequent between 70 and 22 kyr BP, with alternating drier and wetter periods. The final cool and arid period began approximately 22,000 years ago (LGM) and lasted until approximately 14 kyr BP, when rainfall and temperatures increased again, which favored the return of forests. The warmest interglacial period appears to have occurred in the mid‐Holocene (5,600–2,500 years ago), and after a short warm period, the temperatures decreased again and the climate became drier.

From the cpDNA haplotype network (Figure 1), we generated population connectivity maps by summing the least‐cost path (LCP) among haplotypes from different localities. The LCP is based on the idea that resistance to movement can be mapped by assigning each cell in a map a relative “weighted distance” or “cost” of moving across that cell (Chan et al., 2011). The cell “cost” is determined by the habitat characteristics of the cell and is evaluated by calculating, for each cell, the cumulative weighted distance between the cell in question and two designated source areas. This analysis results in a map that shows the relative linkage value across the landscape (where routes through the landscape encounter more or fewer landscape barriers) between the two source areas (Singleton, Gaines, & Lehmkuhl, 2002). The combined interpretation of genetic population connectivity results and the LCP maps highlight areas and corridors that may have been a source population or historical refuge (Chan et al., 2011).

Using SDMtoolbox 1.1a (Brown, 2014) in ArcMap 10.2.2 (ESRI 2011), with the “Create Friction Layer > Invert SDM tool,” we first inverted species distribution predictions that had previously been generated under current and palaeoclimatic models (mid‐Holocene, LGM, and LIG). Inverted species distribution models produce friction layers with highest values in cells where probabilities of species occurrence are lowest (i.e., highest resistance to colonization). Next, the corridors that minimize the cost of dispersal between sampling localities by following paths of lowest friction were calculated. For these calculations, the “Least‐Cost Corridors and Paths > Pairwise: All Sites tool” was used (default settings).

## 3 RESULTS

In addition to the analysis of the independent segregation hypothesis that showed 6.5% of locus combinations significantly deviated from *p *= .05, no pairs of loci were found to be in significant genotypic disequilibrium after Bonferroni's correction (*p *< .0018). The presence of null alleles was detected for all nuclear loci and populations ranging from 0 to 4 and 0 to 6, when considering the number of loci per population and the number of populations per locus, respectively (Table S2).

### 3.1 Nuclear and plastid genetic diversity

We identified a total of 193 alleles for the set of nSSRs. The mean number of alleles was 44 per population and ranged from 10 to 95 (Table 2). The observed values of allelic richness for the different populations varied from 1.24 to 11.05 (Table 2). The population with the highest allelic richness value was VP (11.05), followed by AD (8.97), CC (8.41), and MU (7.69). Private and rare alleles were detected in high frequency for the set of populations, which were 37% and 70%, respectively. Mean genetic diversity at the species level was equal to *H*_*e*_ **= 0.464, but with values highly variable among populations: 0.010 in CA to 0.833 in VP (Table 2). The mean inbreeding coefficient per population was 0.206, reducing to 0.036 when estimated with exclusion of null alleles (Table 2).

For the cpSSRs, we identified from two to five alleles per locus, whose genetic combinations resulted in 11 different haplotypes (H1–H11, Table 2 and Figure 1). Although the high estimation of total haplotypic diversity (*h *= 0.951), the mean haplotypic diversity was low (*h* = 0.064) due to the high frequency of fixed haplotypes in populations (Table 2, Figure 1). In the haplotype network, it was possible to identify two haplogroups, one with eight haplotypes and the other with three haplotypes (Figure 1a). H3 identified in EC showing the higher number of mutational steps in relation to the other haplotypes (Figure 1A).

### 3.2 Distribution of genetic variability and phylogeographic pattern

High and significant genetic divergences were estimated among populations for both nuclear and plastid DNA by AMOVA (Φ_ST(*n*)_ = 0.463 and Φ_ST(plastid)_ = 0.961, respectively, *p *< .001). The permutation *R*_ST_ test revealed that genetic divergence between distinct alleles of nuclear genome is related to geographical separation, as the *R*_ST_ value was larger than both *F*_ST_ and *pR*_ST_ (0.656 > 0.487 > 0.016, respectively). However, for the plastid genome, it was not possible to identify a clear phylogeographic signal because *N*_ST_ was very similar to *F*_ST_ (0.975 ≅ 0.960).

**Figure 2:**
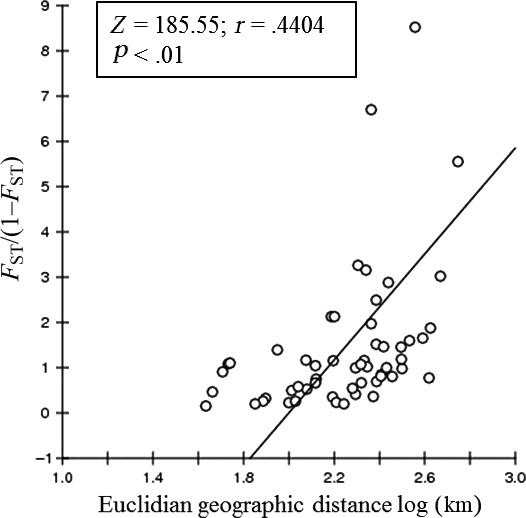
Mantel's test between *F*_ST_/(1–*F*_ST_) and Euclidian geographic distance log (Km)

**Figure 3:**
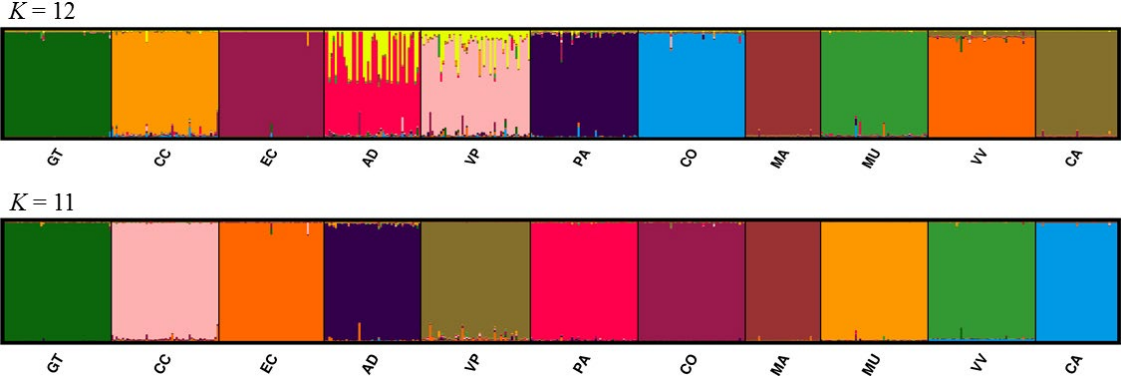
Proportion of multilocus genotype of *Encholirium horridum* L.B.Sm. for *K *=* *12 and *K *=* *11 genetic groups detected by the software STRUCTURE. Distinct colors represent distinct genetic groups. See Table 1 for abbreviations of populations

High and significant isolation by distance for nSSRs were detected using *F*_ST_ values (*Z *=* *185.55, *r *=* *.4404, *p *<* *.01; Figure 2). However, no IBD was detected for cpDNA genome (*Z *=* *6,971.24, *r *=* *−.0268, *p *=* *.4247). Bayesian clustering of groups of individuals revealed 12 and 11 genetic clusters (i.e., populations), considering no a priori population localities and with posterior information of the identity of populations, respectively (Figure 3, Fig. S1). The almost total absence of genetic admixture among populations was evident, especially in the *K* = 11 barplot (Figure 3).

### 3.3 Estimates of gene flow

Values of immigration and emigration rates among populations based on the nSSRs were very low (Table 3), corroborating the strong genetic structure revealed from previous analyses (AMOVA and Bayesian clustering). Both short‐ and long‐term estimates were not statistically significant among populations (Table S3). No pairs of populations showed significant asymmetric short‐term estimates of gene flow, as seen by the overlapping of 95% confidence intervals. In fact, short‐term estimate values were quite similar among the population set, varying from 0.0053 to 0.0074 (*SD* ± 0.0006). Estimates of long‐term gene flow were more variable, ranging from 0.0254 to 0.0001 (±0.047), and only four population pairs showed asymmetric estimates (Table S3). Short‐ and long‐term bidirectional estimates of gene flow across population pairs were not significantly correlated (Spearman's ρ = +.001, *p *=* *.95).

**Table 3:** Summary of net immigration and emigration rates among populations of *Encholirium horridum* L.B.Sm in Sugar Loaf Land, Brazil

Population	Total emigration		Total immigration		Net emigration^a^	
	Short term	Long term	Short term	Long term	Short term	Long term
GT	0.055	0.018	0.058	0.018	−0.003	+0.000
CC	0.058	0.070	0.058	0.095	−0.001	−0.025
EC	0.056	0.049	0.061	0.023	−0.005	+0.026
AD	0.059	0.066	0.059	0.073	+0.001	−0.007
VP	0.055	0.056	0.058	0.099	−0.003	−0.043
PA	0.055	0.050	0.059	0.045	−0.003	+0.005
CO	0.055	0.051	0.058	0.054	−0.004	−0.003
MA	0.072	0.041	0.057	0.031	+0.016	+0.010
MU	0.055	0.066	0.058	0.066	−0.004	+0.000
VV	0.054	0.076	0.058	0.053	−0.004	+0.023
CA	0.068	0.044	0.057	0.030	+0.011	+0.015

See Table 1 for the names of the populations.

a Net emigration = sum of all emigration rates minus the sum of all immigration rates.

### 3.4 Distribution and dispersal corridors across time periods

**Figure 4:**
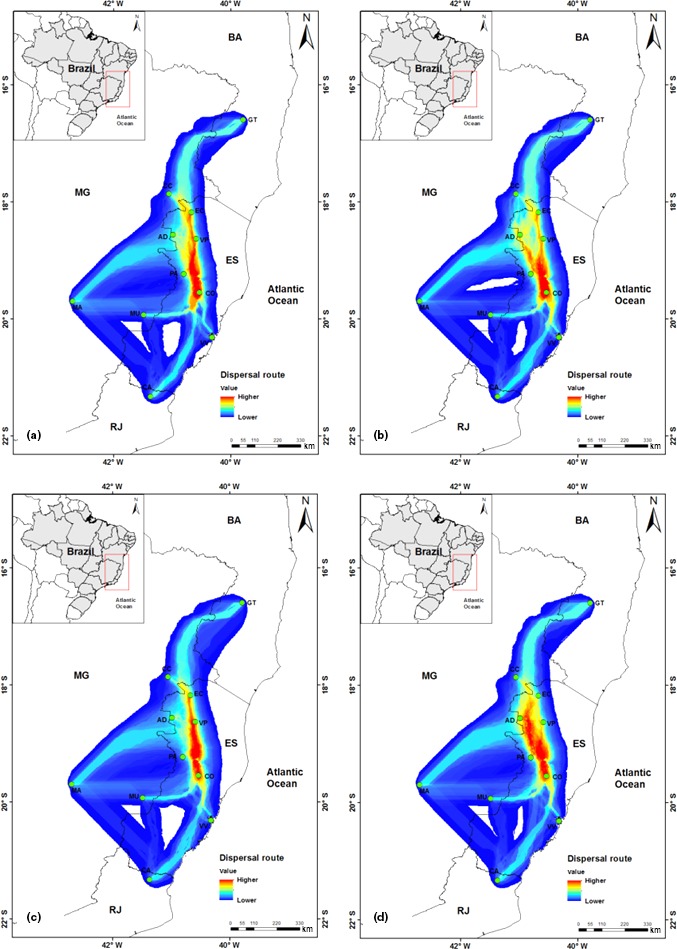
Potential dispersal routes of *Encholirium horridum* L.B.Sm in Sugar Loaf Land across four time periods, based on plastid genome microsatellite markers. (a) Last Interglacial (~120 kyr BP), (b) Last Glacial Maximum (~21 kyr BP), (c) mid‐Holocene (~6 kyr BP), and (d) current conditions

Based on the cpDNA results, putative dispersal routes in Sugar Loaf Land across cycles of interglaciation and glaciations events in the Quaternary period revealed that *E. horridum* population connectivity changed little during climatic fluctuations (Figure 4). Actual climatic conditions seem to be the most propitious to species genetic exchange. Areas with higher environmental suitability and presumable genetic connectivity correspond to inselberg landscapes in Espírito Santo State. These areas, characterized by high inselberg concentration, are also the core area of distribution of *E. horridum* populations.

## 4 DISCUSSION

### 4.1 Patterns of genetic diversity and gene flow among inselbergs

Low gene flow, prolonged isolation, and persistence seems to be a common pattern to populations located in inselberg landscapes around the world (Barbará et al., 2007; Boisselier‐Dubayle et al., 2010; Byrne & Hopper, 2008; Millar et al., 2013; Pinheiro et al., 2011, 2014; Tapper et al., 2014a,b). Random genetic drift and inbreeding have been suggested as main drivers of plant diversification and evolution on terrestrial islands due to limited connectivity among disjunct populations (Millar et al., 2013; Pinheiro et al., 2011, 2014; Tapper et al., 2014a). For example, in extreme cases of isolation, within‐inselberg interspecific gene flow between congeneric species has been found to be higher than intraspecific connectivity between different inselbergs (Barbará et al., 2007; Palma‐Silva et al., 2011). Also, persistence of species on granite outcrops indicates that these outcrops must provide heterogeneity and refugial opportunities (Tapper et al., 2014b) as long as there is a certain degree of connectivity among populations. For instance, genetic diversity indicated that gene flow was sufficient to largely counteract any negative genetic effects of inbreeding and random genetic drift in inselberg populations of *Acacia woodmaniorum* (Millar et al., 2013).

*Encholirium horridum* showed, on average, median genetic diversity but demonstrated a great range in values among populations (Table 2). We observed a tendency for peripheral populations, such as GT, MA, and CA, to have a lower diversity. These populations are located on geographically isolated inselbergs in the landscape. Decline in genetic diversity accompanied by increased differentiation among populations from the center of a species’ geographic distribution to its periphery is a frequent pattern across plants and animals (Eckert, Samis, & Lougheed, 2008). Isolated populations may experience declines in their sizes resulting in a loss of genetic diversity due to the effects of genetic drift and suffer from the Allee effects (Forsyth, 2003; Groom, 1998). Furthermore, the landscape morphology of northwestern Espírito Santo and western Minas Gerais is characterized by a concentration of inselbergs in chain formations. In these landscapes, *E. horridum* populations are found geographically closer to one another, potentially allowing more genetic connectivity and this is precisely where populations with the highest genetic diversity are located.

In face of the detection of high genetic diversity and private alleles in some *E. horridum* populations, and that strong genetic structure was detected in all of the analyses, we suggest there has been long‐term persistence of the populations across time. The stability of potential dispersal routes also reinforces this pattern. Although no clear phylogeographic structure was evident in the results, considering the almost complete fixation of haplotypes, we also suggest that seed dispersal among inselbergs is an occasional phenomenon and the founder effect seems to be an important process in the formation of new populations. Furthermore, the relationships between AMOVA values estimated for both genomes was noteworthy (Φ_ST(*n*)_ = 0.463, Φ_ST(plastid)_ = 0.961; *p *<* *.001). Hamilton and Miller (2002) highlighted that, in the infinite island model, when rates of pollen and seed gene flow are equal, seed dispersal will contribute twice as much as pollen dispersal to the size of genetic neighborhoods (*F*_ST(plastid)_ ~ 2 *F*_ST(*n*)_). This demonstrates that, in *E. horridum,* seed gene flow can be a more important determinant of deme size and subdivision among populations than pollen gene flow. In fact, a strong genetic structure of cpDNA has been verified in other species endemic to granitic inselbergs (Palma‐Silva et al., 2011; Pinheiro et al., 2014; Tapper et al., 2014b).

### 4.2 Biogeographic patterns on Atlantic Forest granitic inselbergs

In Brazil, during the LGM, the retraction of seasonally dry tropical forests occurred and, consequently, Cerrado areas expanded until the mid‐Holocene (Werneck et al., 2011, 2012). In the Cerrado, lower plateaus and peripheral depressions were much drier than today and, probably, dominated by more xeric‐adapted vegetation expanded from the Cerrado, Caatinga, and other colder/drier biomes in southern South America (Ab'Sáber, 2003; Werneck, 2011). The orogenic scenario of inselberg formation in association with these paleovegetation dynamics may have contributed to an enhancement in the degree of isolation of *E. horridum* populations, making them more susceptible to the effects of genetic drift, which lead to accumulation of mutations. The heterogeneity of granite outcrops may provide greater opportunities for contraction to favorable habitats (localized refugia) during periods of unfavorable conditions, with subsequent expansion if conditions improved (Tapper et al., 2014b).

Diversification times of Cerrado woody lineages span the late Miocene to Pliocene (9.8–0.4 million years ago, Ma), with most lineages being <4 Myr old (Simon, Grether, Queiroz, Skema, & Pennington, 2009). Recent phylogenies of Bromeliaceae have proposed the invasion of dry, rocky sites by the *Dyckia*‐*Encholirium* group from the Central Andes through the interior of the Brazilian Shield to the Horn of Brazil c. 8.5 Ma, with a subsequent divergence between genera c. 2.4 Ma (Givnish, Milliam, Berry, & Systma, 2007; Givnish et al., 2011, 2014), at the beginning of the Pleistocene. The eastern portion of Espírito Santo State may have been the initial region of Atlantic Forest granitic inselberg colonization, which occurred during expansion events of open vegetation from Minas Gerais State. For *Hoffmann seggella*, a rupiculous orchid species, Antonelli, Verola, Parisod, and Gustafsson (2010) suggest that the rapid radiation around the Middle/Late Miocene (11–14 Mya) began in the region of southern Minas Gerais, which acted as a main source area for several independent range expansions north and east via episodic corridors to northern Minas Gerais, Espírito Santo, Rio de Janeiro, and Bahia. Later, Pleistocene forest expansion events would have caused the fragmentation of populations and a consequent diversification of lineages. In the same way, Saraiva, Mantovani, and Forzza (2015) identified a clade, the so‐called *Pitcarnia azouri* group, formed by several taxa endemic to granitic inselbergs that are morphologically similar and difficult to differentiate. It is possible that this group may have undergone a more recent radiation, spreading geographically followed by becoming isolated on inselbergs (Saraiva et al., 2015). However, for some *Alcantarea* species from xeric environments (inselbergs and quartzitic outcrops), a reverse biogeographic pattern of colonization has been suggested: Occupation of the rocky savannah‐like vegetation has occurred more than once from Atlantic Forests ancestors (Versieux et al., 2012).

### 4.3 *Encholirium horridum* in the light of the Ocbil theory

Ocbils are defined across three continuously varying axes of landscape age, disturbance regimes, especially relative climatic buffering, and nutrient impoverishment of soils (Hopper, 2009; Hopper et al., 2016). Considering the key hypotheses of Ocbil theory (see Hopper, 2009), we can highlight some interesting points. The strong genetic structure found in both nuclear and plastidial genomes is in consonance with the first prediction, which enunciates the reduced dispersibility and strong differentiation of populations. Restricted pollen flow by paternity analysis was also detected in *E. horridum* (Hmeljevski et al., 2015). The second hypothesis, which predicts the elevated persistence of lineages and long‐lived individuals, is supported by the evidence of the long persistence of populations across time based on genetic data and potential dispersal routes. Still, besides the absence of clonality in *E. horridum*, individuals are perennial and have slow growth (Hmeljevski et al., 2015). Other results, such as mean median genetic diversity of populations—probably purging of deleterious recessive genes (see Hmeljevski et al., 2015)—pollination mediated by vertebrates (Hmeljevski, 2013), and higher importance of pollen dispersal for genetic exchange than dispersal by seeds, are indicative of the occurrence of the James Effect (i.e., the retention of heterozygosity by selection mechanisms; third hypothesis). Lastly, the possible invasion of Atlantic Forest granitic inselbergs by routes from drier surrounding environments is in accordance with the fourth (Semiarid Cradle Hypothesis) prediction of the Ocbil theory. Therefore, based on this set of evidence, *E. horridum* is an Ocbil organism.

In the current scenario, genetic analyses highlighted the low genetic connectivity and long‐term persistence of *E. horridum* populations, reinforcing the geographic isolation of species restricted to inselbergs. Phenomena such as the founder effect and genetic drift appear to have been very important in shaping the current diversity and genetic structure observed in both genomes. The conjoint analysis of results obtained from the species distribution models and genetic markers point to the likely stability of the species in its core distribution area during Quaternary climatic oscillations. Finally, the set of results obtained from this and previous studies of *E. horridum* strongly suggests this species as an Ocbil organism. Although numerous and dense populations can be found in nature, the set of results enhance the genetic singularity of each population and the clear need of the in situ conservation of all of them. Nearly one‐third of the terrestrial biodiversity hotspots contain large or small Ocbil landscapes that deserve special treatment in conservation and restoration efforts (Hopper et al., 2016). Inselbergs are recognized features of high endemism and constitute important areas of xeric species diversity in the Atlantic Forest. Nowadays, these inselberg landscapes are mostly surrounded by agricultural operations and pasture and are not included in any integral protected areas. Most Brazilian mountain areas still lack specific conservation and management plans and public policies are extremely essential and urgent to conserve these peculiar environments.

## CONFLICT OF INTEREST

None declared.

## References

[ece33038-bib-0001] Ab'Sáber, A. N. (2003). O domínio dos Cerrados In Ab'Sáber A. N. (Ed.), Domínios de natureza no Brasil: Potencialidades paisagísticas (pp. 115–136). São Paulo, SP: Ateliê Editorial.

[ece33038-bib-0002] Alkmim, F. F. (2015). Geological background: A tectonic panorama of Brazil In Vieira B. C., Salgado A. A. R., & Santos L. J. C. (Eds.), Landscapes and landforms of Brazil (pp. 9–18). World Geomorphological Landscapes series, Dordrecht, Heidelberg, New York, London: Springer.

[ece33038-bib-0003] Antonelli, A. , Verola, C. , Parisod, C. , & Gustafsson, A. L. (2010). Climate cooling promoted the expansion and radiation of a threatened group of South American orchids (Epidendroideae: Laeliinae). Biological Journal of the Linnean Society, 100, 597–607.

[ece33038-bib-0004] Bandelt, H.‐J. , Forster, P. , & Röhl, A. (1999). Median‐Joining networks for inferring intraspecific phylogenies. Molecular Biology and Evolution, 16, 37–48. 10331250 10.1093/oxfordjournals.molbev.a026036

[ece33038-bib-0005] Barbará, T. , Martinelli, G. , Fay, M. F. , Mayo, S. J. , & Lexer, C. (2007). Population differentiation and species cohesion in two closely related plants adapted to neotropical high‐altitude ‘inselbergs’, *Alcantarea imperialis* and *Alcantarea geniculata* (Bromeliaceae). Molecular Ecology, 16, 1981–1992. 17498226 10.1111/j.1365-294X.2007.03272.x

[ece33038-bib-0006] Beerli, P. , & Felsenstein, J. (1999). Maximum‐likelihood estimation of migration rates and effective population numbers in two populations using a coalescent approach. Genetics, 152, 763–773. 10353916 10.1093/genetics/152.2.763 PMC1460627

[ece33038-bib-0007] Beerli, P. , & Felsenstein, J. (2001). Maximum likelihood estimation of a migration matrix and effective population sizes in n subpopulations by using a coalescent approach. Proceedings of the National Academy of Sciences of the United States of America, 98, 4563–4568. 11287657 10.1073/pnas.081068098 PMC31874

[ece33038-bib-0008] Behling, H. (2002). South and southeast Brazilian grasslands during Late Quaternary times: A synthesis. Palaeogeography, Palaeoclimatology, Palaeoecology, 177, 19–27.

[ece33038-bib-0009] Boisselier‐Dubayle, M. C. , Leblois, R. , Samadi, S. , Lambourdière, J. , & Sarthou, C. (2010). Genetic structure of the xerophilous bromeliad *Pitcairnia geyskesii* on inselbergs in French Guiana—A test of the forest refuge hypothesis. Ecography, 33, 175–184.

[ece33038-bib-0010] Brown, J. L. (2014). SDMtoolbox: A python‐based GIS toolkit for landscape genetic, biogeographic and species distribution model analyses. Methods in Ecology and Evolution, 5, 694–700. 10.7717/peerj.4095 PMC5721907 29230356

[ece33038-bib-0011] Bueno, M. L. , Pennington, R. T. , Dexter, K. G. , Kamino, L. H. Y. , Pontara, V. , Neves, D. M. , … Oliveira‐Filho, A. T. (2016). Effects of Quaternary climatic fluctuations on the distribution of Neotropical savanna tree species. Ecography, 39, 001–012.

[ece33038-bib-0012] Byrne, M. , & Hopper, S. (2008). Granite outcrops as ancient islands in old landscapes: Evidence from the phylogeography and population genetics of *Eucalyptus caesia* (Myrtaceae) in Western Australia. Biological Journal of the Linnean Society, 93, 177–188.

[ece33038-bib-0013] Carnaval, A. C. , Hickerson, M. J. , Haddad, C. F. B. , Rodrigues, M. T. , & Moritz, C. (2009). Stability predicts genetic diversity in the Brazilian Atlantic forest hotspot. Science, 323, 785–789. 19197066 10.1126/science.1166955

[ece33038-bib-0014] Carnaval, A. C. , & Moritz, C. (2008). Historical climate modeling predicts patterns of current biodiversity in the Brazilian Atlantic forest. Journal of Biogeography, 35, 1187–1201.

[ece33038-bib-0015] Chan, L. M. , Brown, J. L. , & Yoder, A. D. (2011). Integrating statistical genetic and geospatial methods brings new power to phylogeography. Molecular Phylogenetics and Evolution, 59, 523–537. 21352934 10.1016/j.ympev.2011.01.020

[ece33038-bib-0016] Collevatti, R. G. , Terribile, L. C. , Lima‐Ribeiro, M. S. , Nabout, J. C. , Oliveira, G. , Rangel, T. F. , … Diniz‐Filho, J. A. F. (2012). A coupled phylogeographical and species distribution modelling approach recovers the demographical history of a Neotropical seasonally dry forest tree species. Molecular Ecology, 21, 5845–5863. 23094833 10.1111/mec.12071

[ece33038-bib-0017] Dantas, G. P. M. , Sari, E. H. R. , Cabanne, G. S. , Pessoa, R. O. , Marini, M. A. , Miyaki, C. Y. , & Santos, F. R. (2015). Population genetic structure of the Atlantic Forest endemic *Conopophaga lineata* (Passeriformes: Conopophagidae) reveals a contact zone in the Atlantic Forest. Journal of Ornithology, 156, 85–99.

[ece33038-bib-0018] Dormann, C. F. , Elith, J. , Bacher, J. S. , Buchmann, C. , Carl, G. , Carré, G. , … Lautenbach, S. (2013). Collinearity: A review of methods to deal with it and a simulation study evaluating their performance. Ecography, 36, 027–046.

[ece33038-bib-0019] Duminil, J. , Heuertz, M. , Doucet, J.‐L. , Bourland, N. , Cruaud, C. , Gavory, F. , … Hardy, O. J. (2010). CpDNA‐based species identification and phylogeography: Application to African tropical tree species. Molecular Ecology, 19, 5469–5483. 21091558 10.1111/j.1365-294X.2010.04917.x

[ece33038-bib-0020] Eckert, C. G. , Samis, K. E. , & Lougheed, S. C. (2008). Genetic variation across species’ geographical ranges: The central–marginal hypothesis and beyond. Molecular Ecology, 17, 1170–1188. 18302683 10.1111/j.1365-294X.2007.03659.x

[ece33038-bib-0021] ESRI (2011). ArcGIS desktop: Release 10. Redlands, CA: Environmental Systems Research Institute.

[ece33038-bib-0101] Evanno, G. , Regnaut, S. , & Goudet, J. (2005). Detecting the number of clusters of individuals using the software STRUCTURE: a simulation study. Molecular Ecology, 14, 2611–2620. 15969739 10.1111/j.1365-294X.2005.02553.x

[ece33038-bib-0022] Excoffier, L. , & Lischer, H. E. L. (2010). Arlequin suite version 3.5: A new series of programs to perform population genetics analyses under Linux and Windows. Molecular Ecology Resources, 10, 564–567. 21565059 10.1111/j.1755-0998.2010.02847.x

[ece33038-bib-0023] Forsyth, S. A. (2003). Density‐dependent seed set in the Haleakala silversword: Evidence for an Allee effect. Oecologia, 136, 551–557. 12783298 10.1007/s00442-003-1295-3

[ece33038-bib-0024] Forzza, R. C. (2005). Revisão taxonômica de *Encholirium* Mart. ex Schult. & Schult. F. (Pitcairnioideae–Bromeliaceae). Boletim Botânico da Universidade de São Paulo, 23, 1–49.

[ece33038-bib-0025] Forzza, R. C. , Costa, A. F. , Leme, E. M. C. , Versieux, L. M. , Wanderley, M. G. L. , Louzada, R. B. , … Moraes, M. A. (2013). Bromeliaceae In (org by Martinelli G. & Moraes M. A.), The Red Book of Brazilian Flora (pp. 315–396). Rio de Janeiro, RJ: Rio de Janeiro Botanical Garden Research Institute. Andrea Jakobsson.

[ece33038-bib-0026] Forzza, R. C. , & Leme, E. M. C. (2015). Three new species of *Encholirium* (Bromeliaceae) from eastern Brazil. Phytotaxa, 227, 13.

[ece33038-bib-0027] Gent, P. R. , Danabasoglu, G. , Donner, L. J. , Holland, M. M. , Hunke, E. C. , Jayne, S. R. , … Zhang, M. (2011). The Community Climate System Model Version 4. Journal of Climate, 24, 4973–4991.

[ece33038-bib-0028] Givnish, T. J. , Barfuss, M. H. J. , Van Ee, B. , Riina, R. , Schulte, K. , Horres, R. , … Systma, K. J. (2014). Adaptive radiation, correlated and contingent evolution, and net species diversification in Bromeliaceae. Molecular Phylogenetics and Evolution, 71, 55–78. 24513576 10.1016/j.ympev.2013.10.010

[ece33038-bib-0029] Givnish, T. J. , Barfuss, M. H. J. , Van Ee, B. , Riina, R. , Schulte, K. , Horres, R. , … Sytsma, K. J. (2011). Phylogeny, adaptive radiation, and historical biogeography in Bromeliaceae: Insights from an eightlocus plastid phylogeny. American Journal of Botany, 98, 872–895. 21613186 10.3732/ajb.1000059

[ece33038-bib-0030] Givnish, T. J. , Milliam, K. C. , Berry, P. E. , & Systma, K. J. (2007). Phylogeny, adaptive radiation, and historical biogeography of Bromeliaceae inferred from *ndh*f sequence data. Aliso, 23, 3–26.

[ece33038-bib-0031] Goudet, J. (1995). FSTAT (Version1.2): A computer program to calculate *F*‐statistics. Journal of Heredity, 86, 485–486.

[ece33038-bib-0032] Gradim, C. , Roncato, J. , Pedrosa‐Soares, A. C. , Cordani, U. , Dussin, I. , Alkmim, F. F. , … Babinski, M. (2014). The hot back‐arc zone of the Araçuaí orogen, Eastern Brazil: From sedimentation to granite generation. Brazilian Journal of Geology, 44, 155–180.

[ece33038-bib-0033] Groom, M. J. (1998). Allee effects limit population viability of an annual plant. The American Naturalist, 151, 487–496. 10.1086/286135 18811371

[ece33038-bib-0102] Hamilton, M. B. , & Miller, J. R. (2002). Comparing relative rates of pollen and seed gene flow in the island model using nuclear and organelle measures of population Structure. Genetics, 62, 1897–1909. 10.1093/genetics/162.4.1897 PMC1462371 12524358

[ece33038-bib-0034] Hardy, O. J. , Charbonnel, N. , Fréville, H. , & Heuertz, M. (2003). Microsatellite allele sizes: A simple test to assess their significance on genetic differentiation. Genetics, 163, 1467–1482. 12702690 10.1093/genetics/163.4.1467 PMC1462522

[ece33038-bib-0035] Hardy, O. , & Vekemans, X. (2002). SPAGeDI: A versatile computer program to analyze spatial genetic structure at the individual or population levels. Molecular Ecology Notes, 2, 618–620.

[ece33038-bib-0036] Hijmans, R. J. , Cameron, S. E. , Parra, J. L. , Jones, P. G. , & Jarvis, A. (2005). Very high resolution interpolated climate surfaces for global land areas. International Journal of Climatology, 25, 1965–1978.

[ece33038-bib-0038] Hmeljevski, K. V. , Ciampi, M. B. , Baldauf, C. , Reis, M. S. , & Forzza, R. C. (2013). Development of SSR markers for *Encholirium horridum* (Bromeliaceae) and transferability to other Pitcairnioideae. Applications in Plant Sciences, 1, 1200445. 10.3732/apps.1200445 PMC4105294 25202537

[ece33038-bib-0039] Hmeljevski, K. V. , Reis, M. S. , & Forzza, R. C. (2015). Patterns of gene flow in *Encholirium horridum* L.B.Sm., a monocarpic species of Bromeliaceae from Brazil. Journal of Heredity, 106, 93–101. 25472982 10.1093/jhered/esu067

[ece33038-bib-0103] Hmeljevski, K. V. , Wolowski, M. , Forzza, R. C. , & Freitas, L. (2017). High outcrossing rates and short‐distance pollination in a species restricted to granitic inselbergs. Australian Journal of Botany, In press.

[ece33038-bib-0040] Hopper, S. D. (2009). OCBIL theory: Towards an integrated understanding of the evolution, ecology and conservation of biodiversity on old, climatically buffered, infertile landscapes. Plant and Soil, 322, 49–86.

[ece33038-bib-0041] Hopper, S. D. , Silveira, F. A. O. , & Fiedler, P. L. (2016). Biodiversity hotspots and Ocbil theory. Plant and Soil, 403, 167–216.

[ece33038-bib-0042] Howes, B. J. , Brown, J. W. , Gibbs, H. L. , Herman, T. B. , Mockford, S. W. , Prior, K. A. , & Weatherhead, P. J. (2009). Directional gene flow patterns in disjunct populations of the black ratsnake (*Pantheropis obsoletus*) and the Blanding's turtle (*Emydoidea blandingii*). Conservation Genetics, 10, 407–417.

[ece33038-bib-0043] Jensen, J. L. , Bohonak, A. J. , & Kelley, S. T. (2005). Isolation by distance, web service. Retrieved from http://ibdws.sdsu.edu/ 10.1186/1471-2156-6-13 PMC1079815 15760479

[ece33038-bib-0044] Keenan, K. , McGinnity, P. , Cross, T. F. , Crozier, W. W. , & Prodohl, P. A. (2013). diveRsity: An R package for the estimation and exploration of population genetics parameters and their associated errors. Methods in Ecology and Evolution, 4, 782–788.

[ece33038-bib-0045] Kopelman, N. M. , Mayzel, J. , Jakobsson, M. , Rosenberg, N. A. , & Mayrose, I. (2015). CLUMPAK: A program for identifying clustering modes and packaging population structure inferences across *K* . Molecular Ecology Resources, 15, 1179–1191. 25684545 10.1111/1755-0998.12387 PMC4534335

[ece33038-bib-0046] Krapp, F. , Pinnangé, D. S. B. , Benko‐Iseppon, A. M. , Leme, E. M. C. , & Weising, K. (2014). Phylogeny and evolution of *Dyckia* (Bromeliaceae) inferred from chloroplast and nuclear sequences. Plant Systematics and Evolution, 300, 1591–1614.

[ece33038-bib-0047] Lima, G. M. P. , & Corrêa‐Gomes, L. C. (2015). Itatim geomorphological site: Largest concentration of inselbergs in Brazil In Vieira B. C., Salgado A. A. R., & Santos L. J. C. (Eds.), Landscapes and landforms of Brazil (pp. 371–380). World geomorphological landscapes, Dordrecht: Springer Science+Business Media https://doi.org/10.1007/978%e2%80%9394%e2%80%93017-8023-0

[ece33038-bib-0048] Lowe, W. H. , & Allendorf, F. W. (2010). What can genetics tell us about population connectivity? Molecular Ecology, 19, 3038–3051. 20618697 10.1111/j.1365-294X.2010.04688.x

[ece33038-bib-0049] Lüttge, U. (2008). Physiological ecology of tropical plants. Berlin: Springer‐Verlag.

[ece33038-bib-0050] Manel, S. , & Holderegger, R. (2013). Ten years of landscape genetics. Trends in Ecology and Evolution, 28, 614–621. 23769416 10.1016/j.tree.2013.05.012

[ece33038-bib-0051] Manel, S. , Schwartz, M. K. , Luikart, G. , & Taberlet, P. (2003). Landscape genetics: Combining landscape ecology and population genetics. Trends in Ecology and Evolution, 18, 189–197.

[ece33038-bib-0052] Martins, F. M. (2011). Historical biogeography of the Brazilian Atlantic forest and the Carnaval‐Moritz model of Pleistocene refugia: What do phylogeographical studies tell us? Biological Journal of the Linnean Society, 104, 499–509.

[ece33038-bib-0053] Melo, W. A. , Lima‐Ribeiro, M. S. , Terribile, L. C. , & Collevatti, R. G. (2016). Coalescent simulation and paleodistribution modeling for *Tabebuia rosealba* do not support South American dry forest refugia hypothesis. PLoS One, 11(7), e0159314. 27458982 10.1371/journal.pone.0159314 PMC4961443

[ece33038-bib-0054] Menezes, R. G. , & Sampaio, P. R. A. (2012). Rochas Ornamentais no Noroeste do Estado do Espírito Santo. Informe de Recursos Minerais do Brasil, Série Rochas e Minerais Industriais, 8.

[ece33038-bib-0055] Millar, M. A. , Coates, D. J. , & Byrne, M. (2013). Genetic connectivity and diversity in inselberg populations of *Acacia woodmaniorum*, a rare endemic of the Yilgarn Craton banded iron formations. Heredity, 111, 437–444. 23860233 10.1038/hdy.2013.66 PMC3806024

[ece33038-bib-0056] Nathan, R. , & Muller‐Landau, H. C. (2000). Spatial patterns of seed dispersal, their determinants and consequences for recruitment. TREE, 15, 278–285. 10856948 10.1016/s0169-5347(00)01874-7

[ece33038-bib-0057] Neto, J. L. S. , Galvani, E. , & Vieira, B. C. (2015). Climates of Brazil: Past and present In Vieira B. C., Salgado A. A. R., & Santos L. J. C. (Eds.), Landscapes and landforms of Brazil (pp. 33–41). World geomorphological landscapes. Dordrecht: Springer Science+Business Media https://doi.org/10.1007/978%e2%80%9394%e2%80%93017-8023-0

[ece33038-bib-0058] Oliveira, P. E. , Behling, H. , Ledru, M. P. , Barberi, M. , Bush, M. , Salgado‐Labouriau, M. L. , … Ybert, R. S. (2005). Paleovegetação e paleoclimas do Quaternário do Brasil In Souza C. R. G., Suguio K., Oliveira M. A. S., & Oliveira P. E. (Eds.), Quaternário do Brasil (pp. 52–74). Ribeirão Preto, SP: Holos Editora.

[ece33038-bib-0059] Otto‐Bliesner, B. L. , Marsha, S. J. , Overpeck, J. T. , Miller, G. H. , Hu, A. X. , & Mem, C. L. I. P. (2006). Simulating arctic climate warmth and icefield retreat in the last interglaciation. Science, 311, 1751–1753. 16556838 10.1126/science.1120808

[ece33038-bib-0060] Palma‐Silva, C. , Lexer, C. , Paggi, G. M. , Barbará, T. , Bered, F. , & Bodanese‐Zanettini, M. H. (2009). Range‐wide patterns of nuclear and chloroplast DNA diversity in *Vriesea gigantea* (Bromeliaceae), a neotropical forest species. Heredity, 103, 503–512. 19738634 10.1038/hdy.2009.116

[ece33038-bib-0061] Palma‐Silva, C. , Wendt, T. , Pinheiro, F. , Barbará, T. , Fay, M. M. , Cozzolino, S. , & Lexer, C. (2011). Sympatric bromeliad species (*Pitcairnia* spp.) facilitate tests of mechanisms involved in species cohesion and reproductive isolation in Neotropical inselbergs. Molecular Ecology, 20, 3185–3201. 21672064 10.1111/j.1365-294X.2011.05143.x

[ece33038-bib-0062] de Paula, L. F. A. , Forzza, R. C. , Neri, A. V. , Bueno, M. L. , & Porembski, S. (2016). Sugar Loaf Land in south‐eastern Brazil: A centre of diversity for mat‐forming bromeliads on inselbergs. Botanical Journal of the Linnean Society, 181, 459–476. https://doi.org/10.1111/boj.12383

[ece33038-bib-0063] Peakall, R. , & Smouse, P. E. (2006). GenAlEx 6: Genetic analysis in Excel. Population genetic software for teaching and research. Molecular Ecology Notes, 6, 288–295. 10.1093/bioinformatics/bts460 PMC3463245 22820204

[ece33038-bib-0064] Peakall, R. , & Smouse, P. E. (2012). GenAlEx 6.5: Genetic analysis in Excel. Population genetic software for teaching and research—An update. Bioinformatics Applications Note, 28, 2537–2539. 10.1093/bioinformatics/bts460 PMC3463245 22820204

[ece33038-bib-0065] Petit, R. J. , El Mousadik, A. , & Pons, O. (1998). Identifying populations for conservation on the basis of genetic markers. Conservation Biology, 12, 844–855.

[ece33038-bib-0066] Phillips, S. J. , & Dudik, M. (2008). Modeling of species distributions with MaxEnt: New extensions and a comprehensive evaluation. Ecography, 31, 161–175.

[ece33038-bib-0067] Pinheiro, F. , Barros, F. , Palma‐Silva, C. , Fay, M. F. , Lexer, C. , & Cozzolino, S. (2011). Phylogeography and genetic differentiation along the distributional range of the orchid *Epidendrum fulgens*: A Neotropical coastal species not restricted to glacial refugia. Journal of Biogeography, 38, 1923–1935.

[ece33038-bib-0068] Pinheiro, F. , Cozzolino, S. , Draper, D. , Barros, F. , Félix, L. P. , Fay, M. F. , & Palma‐Silva, C. (2014). Rock outcrop orchids reveal the genetic connectivity and diversity of inselbergs of northeastern Brazil. BMC Evolutionary Biology, 14, 49 https://doi.org/10.1186/1471-2148-14-49 24629134 10.1186/1471-2148-14-49 PMC4004418

[ece33038-bib-0104] Pons, O. , & Petit, R. J. (1996). Measuring and testing genetic differentiation with ordered vs. unordered alleles. Genetics, 144, 1237–1245. 8913764 10.1093/genetics/144.3.1237 PMC1207615

[ece33038-bib-0069] Porembski, S. (2007). Tropical inselbergs: Habitat types, adaptive strategies and diversity patterns. Revista Brasileira de Botânica, 30, 579–586.

[ece33038-bib-0070] Porembski, S. , & Barthlott, W. (Eds) (2000). Inselbergs: Biotic diversity of isolated rock outcrops in tropical and temperate regions (Vol 146). Ecological studies. Heidelberg, Germany: Springer‐Verlag.

[ece33038-bib-0071] Pritchard, J. K. , Stephens, M. , & Donnelly, P. (2000). Inference of population structure using multilocus genotype data. Genetics, 155, 945–959. 10835412 10.1093/genetics/155.2.945 PMC1461096

[ece33038-bib-0105] Rice, W. R. (1989). Analyzing tables of statistical tests. Evolution, 43, 223–225. 28568501 10.1111/j.1558-5646.1989.tb04220.x

[ece33038-bib-0072] Salgado, A. A. R. , Bueno, G. T. , Diniz, A. D. , & Marent, B. R. (2015). Long‐term geomorphological evolution of Brazilian territory In Vieira B. C., Salgado A. A. R., & Santos L. J. C. (Eds.), Landscapes and landforms of Brazil (pp. 19–31). World geomorphological landscapes. Dordrecht: Springer Science+Business Media https://doi.org/10.1007/978%e2%80%9394%e2%80%93017-8023-0

[ece33038-bib-0073] Saraiva, D. P. , Mantovani, A. , & Forzza, R. C. (2015). Insights into the Evolution of *Pitcairnia* (Pitcairnioideae‐Bromeliaceae), based on morphological evidence. Systematic Botany, 40, 726–736.

[ece33038-bib-0074] Schütz, N. , Krapp, F. , Wagner, N. , & Weising, K. (2016). Phylogenetics of Pitcairnioideae s.s. (Bromeliaceae): Evidence from nuclear and plastid DNA sequence data. Botanical Journal of the Linnean Society, 181, 323–342. https://doi.org/10.1111/boj.12403

[ece33038-bib-0106] Selkoe, K. A. , & Toonen, R. J. (2006). Microsatellites for ecologists: a practical guide to using and evaluating microsatellite markers. Ecology Letters, 9, 615–629. 16643306 10.1111/j.1461-0248.2006.00889.x

[ece33038-bib-0075] Simon, M. F. , Grether, R. , Queiroz, L. P. , Skema, C. , & Pennington, R. T. (2009). Recent assembly of the Cerrado, a neotropical plant diversity hotspot, by *in situ* evolution of adaptations to fire. Proceedings of the National Academy of Sciences of the United States of America, 106, 20359–20364. 19918050 10.1073/pnas.0903410106 PMC2787167

[ece33038-bib-0076] Singleton, P. H. , Gaines, W. L. , & Lehmkuhl, J. F. (2002). Landscape permeability for large carnivores in Washington: A Geographic Information System weighted‐distance and least‐cost corridor assessment. Research Paper PNW‐RP‐549. Portland, OR: U.S. Department of Agriculture, Forest Service, Pacific Northwest Research Station.

[ece33038-bib-0077] Sork, V. L. , & Waits, L. (2010). Contributions of landscape genetics—Approaches, insights, and future potential. Molecular Ecology, 19, 3489–3495. 20723050 10.1111/j.1365-294X.2010.04786.x

[ece33038-bib-0078] Storfer, A. , Murphy, M. A. , Evans, J. S. , Goldberg, C. S. , Robinson, S. , Spear, S. F. , … Waits, L. P. (2007). Putting the ‘landscape’ in landscape genetics. Heredity, 98, 128–142. 17080024 10.1038/sj.hdy.6800917

[ece33038-bib-0079] Storfer, A. , Murphy, M. A. , Spear, S. F. , Holderegger, R. , & Waits, L. P. (2010). Landscape genetics: Where are we now? Molecular Ecology, 19, 3496–3514. 20723061 10.1111/j.1365-294X.2010.04691.x

[ece33038-bib-0080] Szarzynski, J. (2000). Xeric islands: Environmental conditions on insel‐ bergs In Porembski S. & Barthlott W. (Eds.), Inselbergs: Biotic diversity of isolated rock outcrops in tropical and temperate regions (Vol. 146, pp. 37–48). Ecological studies. Heidelberg, Germany: Springer‐Verlag.

[ece33038-bib-0081] Tapper, S.‐L. , Byrne, M. , Yates, C. J. , Keppel, G. , Hopper, S. D. , Niel, K. , … Wardell‐Johnson, G. W. (2014a). Isolated with persistence or dynamically connected? Genetic patterns in a common granite outcrop endemic. Diversity and Distribution, 20, 987–1001.

[ece33038-bib-0082] Tapper, S.‐L. , Byrne, M. , Yates, C. J. , Keppel, G. , Hopper, S. D. , Niel, K. , … Wardell‐Johnson, G. W. (2014b). Prolonged isolation and persistence of a common endemic on granite outcrops in both mesic and semi‐arid environments in south‐western Australia. Journal of Biogeography, 41, 2032–2044.

[ece33038-bib-0083] Taylor, K. E. , Stouffer, R. J. , & Meehl, G. A. (2012). An overview of CMIP5 and the experiment design. Bulletin of the American Meteorological Society, 93, 485–498.

[ece33038-bib-0084] Turchetto‐Zolet, A. C. , Pinheiro, F. , Salgueiro, F. , & Palma‐Silva, C. (2013). Phylogeographic patterns shed light on evolutionary process in South America. Molecular Ecology, 22, 1193–1213. 23279129 10.1111/mec.12164

[ece33038-bib-0086] Udupa, S. M. , & Baum, M. (2001). High mutation rate and mutational bias at (TAA)n microsatellite loci in chickpea (*Cicerarietinum* L.). Molecular Genetics Genomics, 265, 1097–1103. 11523782 10.1007/s004380100508

[ece33038-bib-0107] Van Oosterhout, C. , Hutchinson, W. F. , Wills, D. P. M. , & Shipley, P. (2004). Microchecker: software for identifying and correcting genotyping errors in microsatellite data. Molecular Ecology Notes, 4, 535–538.

[ece33038-bib-0087] Varajão, C. A. C. , & Alkmim, F. F. (2015). Pancas: The kingdom of bornhardts In Vieira B. C., Salgado A. A. R., & Santos L. J. C. (Eds.), Landscapes and landforms of Brazil (pp. 381–388). World geomorphological landscapes. Dordrecht: Springer Science+Business Media https://doi.org/10.1007/978%e2%80%9394%e2%80%93017-8023-0

[ece33038-bib-0088] Versieux, L. M. , Barbará, T. , Wanderley, M. G. L. , Calvente, A. , Fay, M. F. , & Lexer, C. (2012). Molecular phylogenetics of the Brazilian giant bromeliads (*Alcantarea*, Bromeliaceae): Implications for morphological evolution and biogeography. Molecular Phylogenetics and Evolution, 64, 177–189. 22491070 10.1016/j.ympev.2012.03.015

[ece33038-bib-0089] Vitorino, L. C. , Lima‐Ribeiro, M. S. , Terribile, L. C. , & Collevatti, R. G. (2016). Demographical history and palaeodistribution modelling show range shift towards Amazon Basin for a Neotropical tree species in the LGM. BMC Evolutionary Biology, 16, 213. 27737632 10.1186/s12862-016-0779-9 PMC5062830

[ece33038-bib-0090] Weir, B. S. , & Cockerham, C. C. (1984). Estimating *F*‐statistics for the analysis of population structure. Evolution, 38, 1358–1370. 28563791 10.1111/j.1558-5646.1984.tb05657.x

[ece33038-bib-0091] Weising, K. , & Gardner, R. C. (1999). A set of conserved PCR primers for the analysis of simple sequence repeat polymorphisms in chloroplast genomes of dicotyledonous angiosperms. Genome, 42, 9–19. 10207998

[ece33038-bib-0092] Werneck, F. P. (2011). The diversification of eastern South American open vegetation biomes: Historical biogeography and perspectives. Quaternary Science Reviews, 30, 1630–1648.

[ece33038-bib-0093] Werneck, F. , Costa, G. C. , Colli, G. R. , Prado, D. E. , & Sites Jr, J. W. (2011). Revisiting the historical distribution of Seasonally Dry Tropical Forests: New insights based on palaeodistribution modelling and palynological evidence. Global Ecology and Biogeography, 20, 272–288.

[ece33038-bib-0094] Werneck, F. P. , Nogueira, C. , Colli, G. R. , Sites Jr, J. W. , & Costa, G. C. (2012). Climatic stability in the Brazilian Cerrado: Implications for biogeographical connections of South American savannas, species richness and conservation in a biodiversity hotspot. Journal of Biogeography, 39, 1695–1706.

[ece33038-bib-0095] Wilson, G. A. , & Rannala, B. (2003). Bayesian inference of recent migration rates using multilocus genotypes. Genetics, 163, 1177–1191. 12663554 10.1093/genetics/163.3.1177 PMC1462502

[ece33038-bib-0096] Yu, H. , Zhang, Y. , Liu, L. , Qi, W. , Li, S. , & Hu, Z. (2015). Combining the least cost path method with population genetic data and species distribution models to identify landscape connectivity during the late Quaternary in Himalayan hemlock. Ecology and Evolution, 5, 5781–5791. 26811753 10.1002/ece3.1840 PMC4717335

