# Supplementary material for: Do plant populations on distinct inselbergs talk to each other? A case study of genetic connectivity of a bromeliad species in an Ocbil landscape
Source: Ecol Evol. 2017 May 23;7(13):4704–16. doi: 10.1002/ece3.3038 (PMC5496560; doi:10.1002/ece3.3038)
Supplement: Supplementary file 1 [file ECE3-7-4704-s001.docx]

**SUPPORTING INFORMATION**

**Do plant populations on distinct inselbergs talk to each other? A case study of genetic connectivity of a bromeliad species in an Ocbil landscape**

Hmeljevski, Karina Vanessa; Nazareno, Alison Gonçalves; Bueno, Marcelo; Maurício Sedrez dos Reis; Forzza, Rafaela Campostrini

**Table S1.** Selected Bioclimatic variables used for the ecological modelling analyses for *Encholirium horridum* L.B.Sm. in Sugar Loaf Land.

| **Code** | **Description** |
| --- | --- |
| BIO 1 | Annual Mean Temperature |
| BIO 4 | Temperature Seasonality (standard deviation *100) |
| BIO 5 | Max Temperature of Warmest Month |
| BIO 6 | Min Temperature of Coldest Month |
| BIO 7 | Temperature Annual Range (BIO5-BIO6) |
| BIO 10 | Mean Temperature of Warmest Quarter |
| BIO 11 | Mean Temperature of Coldest Quarter |
| BIO 12 | Annual Precipitation |
| BIO 13 | Precipitation of Wettest Month |
| BIO 14 | Precipitation of Driest Month |
| BIO 15 | Precipitation Seasonality (Coefficient of Variation) |
| BIO 16 | Precipitation of Wettest Quarter |
| BIO 17 | Precipitation of Driest Quarter |
| BIO 18 | Precipitation of Warmest Quarter |
| BIO 19 | Precipitation of Coldest Quarter |
| ALTITUDE |  |

**Table S2.**Null alleles (Oosterhout correction) – bold values correspond to loci with presence of null alleles.

|  | GT | CC | EC | AD | VP | PA | CO | MA | MU | VV | CA |
| --- | --- | --- | --- | --- | --- | --- | --- | --- | --- | --- | --- |
| EhA07 | -0.0105 | **0.199** | 0.128 | 0.037 | 0.059 | **0.093** | **0.208** | 0 | 0.037 | -0.093 | 0 |
| EhB09 | **0.1938** | -0.002 | **0.209** | **0.071** | 0.032 | -0.007 | 0.0843 | **0.236** | 0.035 | 0.020 | 0 |
| EhC03 | **0.241** | -0.045 | **0.145** | **0.328** | **0.253** | -0.020 | **0.202** | -0.188 | **0.245** | -0.011 | 0 |
| EhE01 | **0.214** | -0.031 | 0 | -0.111 | **0.194** | 0.0923 | -0.118 | 0 | 0.021 | 0 | 0 |
| EhE02 | 0.058 | 0.006 | -0.010 | **0.246** | **0.189** | **0.295** | 0.100 | 0 | **0.177** | **0.299** | -0.013 |
| EhE11 | 0 | -0.039 | 0 | 0.022 | 0.050 | 0.033 | 0 | 0 | 0.018 | -0.026 | 0 |
| EhG03 | **0.130** | **0.119** | 0 | **0.219** | **0.143** | 0.059 | -0.020 | -0.030 | -0.038 | 0.040 | -0.027 |
| EhG07 | 0 | **0.183** | 0.058 | 0.005 | 0.031 | 0.046 | 0.031 | **0.224** | -0.032 | -0.011 | 0 |
| # loci | 4 | 3 | 2 | 4 | 4 | 2 | 2 | 2 | 2 | 1 | 0 |

**
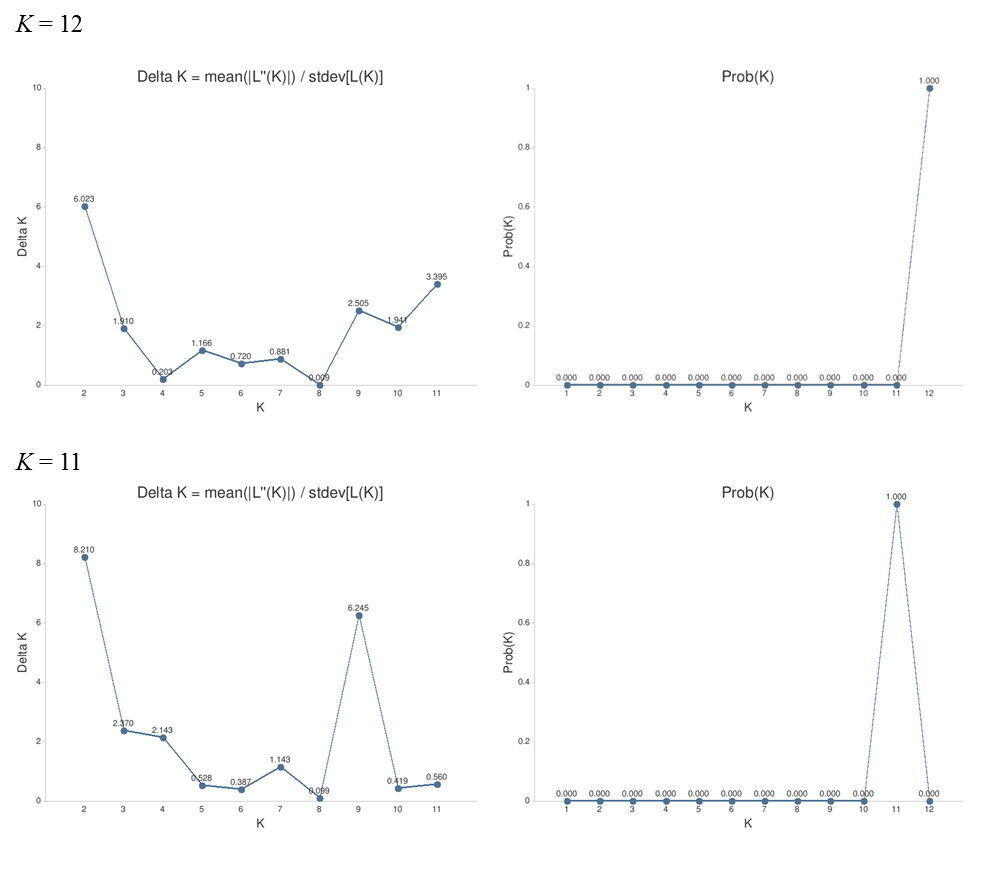
**

**Figure S1.** Determination of the optimum number of groups (K) from the STRUCTURE (Pritchard *et al.,* 2000) analysis for nuclear microsatellite loci in *Encholirium horidum* L.B.Sm. populations sampled in Sugar Loaf Land, Brazil, using the plots of ΔK and Prob(K) against K, as suggested by Evanno *et al.,* (2005).

**Table S3.**Directional gene flow estimates for each population pair of *Encholirium horridum* L.B.Sm.in Sugar Loaf Land, Brazil.

| Population pair | Short-term gene flow (*m*) | | Long-term gene flow (*m*) | |
| --- | --- | --- | --- | --- |
| GT to CC | 0.0055 | (-0.0049-0.0159) | 0.0010 | (-0.0032-0.0051) |
| GT to EC | 0.0054 | (-0.0050-0.0158) | 0.0002 | (-0.0011-0.0014) |
| GT to AD | 0.0054 | (-0.0050-0.0158) | 0.0048 | (-0.0181-0.0278) |
| GT to VP | 0.0055 | (-0.0051-0.0161) | 0.0089 | (-0.0350-0.0527) |
| GT to PA | 0.0054 | (-0.0050-0.0158) | 0.0001 | (-0.0002-0.0005) |
| GT to CO | 0.0056 | (-0.0050-0.0162) | 0.0006 | (-0.0094-0.0107) |
| GT to MA | 0.0055 | (-0.0051-0.0161) | 0.0015 | (-0.0271-0.0301) |
| GT to MU | 0.0054 | (-0.0052-0.0160) | 0.0003 | (-0.0047-0.0053) |
| GT to VV | 0.0056 | (-0.0052-0.0164) | 0.0006 | (-0.0025-0.0037) |
| GT to CA | 0.0056 | (-0.0052-0.0164) | 0.0004 | (-0.0008-0.0015) |
| CC to GT | 0.0055 | (-0.0049-0.0159) | 0.0010 | (-0.0111-0.0130) |
| CC to EC | 0.0083 | (-0.0056-0.0222) | 0.0075 | (-0.6256-0.6405) |
| CC to AD | 0.0055 | (-0.0051-0.0161) | 0.0092 | (-0.0031-0.0216) |
| CC to VP | 0.0054 | (-0.0050-0.0158) | 0.0120 | (-0.0222-0.0462) |
| CC to PA | 0.0056 | (-0.0048-0.0160) | 0.0072 | (-0.6469-0.6613) |
| CC to CO | 0.0055 | (-0.0049-0.0159) | 0.0133 | (-0.3950-0.4217) |
| CC to MA | 0.0054 | (0.0050-0.0158) | 0.0052 | (-0.5310-0.5415) |
| CC to MU | 0.0054 | (-0.0050-0.0158) | 0.0045 | (-1.0095-1.0184) |
| CC to VV | 0.0054 | (-0.0050-0.0158) | 0.0070 | (-0.0007-0.0146) |
| CC to CA | 0.0055 | (-0.0053-0.0163) | 0.0033 | (-0.8592-0.8659) |
| EC to GT | 0.0055 | (-0.0051-0.0161) | 0.0002 | (-0.5550-0.5553) |
| EC to CC | 0.0056 | (-0.0052-0.0164) | 0.0093 | (-0.8754-0.8940) |
| EC to AD | 0.0055 | (-0.0053-0.0163) | 0.0095 | (-0.0007-0.0198) |
| EC to VP | 0.0056 | (-0.0050-0.0162) | 0.0067 | (-0.0854-0.0988) |
| EC to PA | 0.0056 | (-0.0052-0.0164) | 0.0029* | (0.0014-0.0044) |
| EC to CO | 0.0056 | (-0.0054-0.0166) | 0.0069 | (-0.0372-0.0511) |
| EC to MA | 0.0056 | (-0.0050-0.0162) | 0.0005 | (-0.0013-0.0023) |
| EC to MU | 0.0055 | (-0.0051-0.0161) | 0.0029 | (-0.0075-0.0133) |
| EC to VV | 0.0056 | (-0.0050-0.0162) | 0.0088 | (-0.0098-0.0274) |
| EC to CA | 0.0057 | (-0.0053-0.0167) | 0.0014 | (-0.0092-0.0121) |
| AD to GT | 0.0060 | (-0.0056-0.0176) | 0.0048 | (-0.0255-0.0352) |
| AD to CC | 0.0059 | (-0.0057-0.0175) | 0.0080 | (-0.0194-0.0353) |
| AD to EC | 0.0059 | (-0.0055-0.0173) | 0.0026 | (-0.0270-0.0321) |
| AD to VP | 0.0059 | (-0.0055-0.0173) | 0.0072 | (-0.0055-0.0200) |
| AD to PA | 0.0060 | (-0.0056-0.0176) | 0.0086 | (-0.0257-0.0429) |
| AD to CO | 0.0059 | (-0.0055-0.0173) | 0.0072 | (-0.0090-0.0235) |
| AD to MA | 0.0060 | (-0.0054-0.0174) | 0.0046 | (-0.0106-0.0199) |
| AD to MU | 0.0060 | (-0.0056-0.0176) | 0.0145 | (-0.0152-0.0441) |
| AD to VV | 0.0058 | (-0.0054-0.0170) | 0.0019 | (-0.0479-0.0517) |
| AD to CA | 0.0059 | (-0.0055-0.0173) | 0.0068 | (-0.0104-0.0241) |
| VP to GT | 0.0053 | (-0.0049-0.0155) | 0.0089 | (-0.0209-0.0387) |
| VP to CC | 0.0055 | (-0.0051-0.0161) | 0.0070 | (-0.0170-0.0310) |
| VP to EC | 0.0054 | (-0.0050-0.0158) | 0.0016 | (-0.0222-0.0255) |
| VP to AD | 0.0061 | (-0.0055-0.0177) | 0.0049 | (-0.0081-0.0180) |
| VP to PA | 0.0054 | (-0.0050-0.0158) | 0.0060 | (-0.4776-0.4897) |
| VP to CO | 0.0054 | (-0.0048-0.0156) | 0.0095 | (-0.6265-0.6454) |
| VP to MA | 0.0055 | (-0.0053-0.0163) | 0.0034 | (-0.4036-0.4103) |
| VP to MU | 0.0056 | (-0.0050-0.0162) | 0.0078 | (-0.0522-0.0679) |
| VP to VV | 0.0053 | (-0.0049-0.0155) | 0.0024 | (-0.0114-0.0163) |
| VP to CA | 0.0054 | (-0.0052-0.0160) | 0.0041 | (-0.3877-0.3959) |
| PA to GT | 0.0055 | (-0.0051-0.0161) | 0.0001 | (-0.0012-0.0015) |
| PA to CC | 0.0056 | (-0.0050-0.0162) | 0.0135 | (-0.3393-0.3663) |
| PA to EC | 0.0054 | (-0.0050-0.0158) | 0.0005 | (-0.5309-0.5320) |
| PA to AD | 0.0056 | (-0.0052-0.0164) | 0.0117 | (-0.0103-0.0337) |
| PA to VP | 0.0056 | (-0.0054-0.0166) | 0.0114 | (-0.4688-0.4916) |
| PA to CO | 0.0055 | (-0.0053-0.0163) | 0.0062 | (-0.3735-0.3858) |
| PA to MA | 0.0055 | (-0.0051-0.0161) | 0.0006 | (-0.4593-0.4606) |
| PA to MU | 0.0055 | (-0.0051-0.0161) | 0.0045 | (-0.0055-0.0146) |
| PA to VV | 0.0054 | (-0.0050-0.0158) | 0.0009 | (-0.0486-0.0503) |
| PA to CA | 0.0055 | (-0.0051-0.0161) | 0.0004 | (-0.4812-0.4819) |
| CO to GT | 0.0056 | (-0.0052-0.0164) | 0.0006 | (-0.0010-0.0023) |
| CO to CC | 0.0054 | (-0.0048-0.0156) | 0.0163 | (-0.9159-0.9485) |
| CO to EC | 0.0054 | (-0.0050-0.0158) | 0.0053 | (-0.0489-0.0595) |
| CO to AD | 0.0055 | (-0.0049-0.0159) | 0.0066 | (-0.0120-0.0253) |
| CO to VP | 0.0055 | (-0.0051-0.0161) | 0.0121 | (-0.8215-0.8457) |
| CO to PA | 0.0055 | (-0.0051-0.0161) | 0.0022 | (-1.6598-1.6642) |
| CO to MA | 0.0055 | (-0.0053-0.0163) | 0.0006 | (-0.0063-0.0075) |
| CO to MU | 0.0055 | (-0.0049-0.0159) | 0.0024 | (-0.0037-0.0084) |
| CO to VV | 0.0054 | (-0.0052-0.0160) | 0.0043 | (-0.0272-0.0358) |
| CO to CA | 0.0055 | (-0.0051-0.0161) | 0.0005 | (-0.0045-0.0056) |
| MA to GT | 0.0071 | (-0.0066-0.0208) | 0.0015 | (-0.0115-0.0145) |
| MA to CC | 0.0072 | (-0.0067-0.0211) | 0.0097 | (-0.0281-0.0475) |
| MA to EC | 0.0072 | (-0.0065-0.0209) | 0.0002 | (-0.0035-0.0040) |
| MA to AD | 0.0073 | (-0.0068-0.0214) | 0.0059 | (-0.0071-0.0188) |
| MA to VP | 0.0072 | (-0.0067-0.0211) | 0.0105 | (-0.0075-0.0285) |
| MA to PA | 0.0072 | (-0.0065-0.0209) | 0.0015 | (-0.0024-0.0054) |
| MA to CO | 0.0073 | (-0.0066-0.0212) | 0.0015 | (-0.0002-0.0031) |
| MA to MU | 0.0074 | (-0.0065-0.0213) | 0.0021 | (-0.0132-0.0175) |
| MA to VV | 0.0073 | (-0.0068-0.0214) | 0.0072 | (-0.0343-0.0486) |
| MA to CA | 0.0071 | (-0.0066-0.0208) | 0.0011* | (0.0004-0.0018) |
| MU to GT | 0.0055 | (-0.0051-0.0161) | 0.0003 | (-0.0390-0.0396) |
| MU to CC | 0.0055 | (-0.0053-0.0163) | 0.0156 | (-0.0276-0.0589) |
| MU to EC | 0.0055 | (-0.0051-0.0161) | 0.0018 | (-0.0127-0.0162) |
| MU to AD | 0.0055 | (-0.0049-0.0159) | 0.0093 | (-0.0590-0.0776) |
| MU to VP | 0.0055 | (-0.0051-0.0161) | 0.0150 | (-0.0031-0.0332) |
| MU to PA | 0.0055 | (-0.0051-0.0161) | 0.0051 | (-0.0261-0.0363) |
| MU to CO | 0.0054 | (-0.0052-0.0160) | 0.0014 | (-0.0332-0.0359) |
| MU to MA | 0.0055 | (-0.0051-0.0161) | 0.0038 | (-0.0179-0.0255) |
| MU to VV | 0.0054 | (-0.0050-0.0158) | 0.0115 | (-0.0608-0.0837) |
| MU to CA | 0.0054 | (-0.0050-0.0158) | 0.0019 | (-0.0020-0.0058) |
| VV to GT | 0.0054 | (-0.0052-0.0160) | 0.0006 | (-0.0044-0.0056) |
| VV to CC | 0.0054 | (-0.0052-0.0160) | 0.0011 | (-0.0516-0.0538) |
| VV to EC | 0.0055 | (-0.0051-0.0161) | 0.0029 | (-0.0565-0.0622) |
| VV to AD | 0.0055 | (-0.0051-0.0161) | 0.0066 | (-0.0076-0.0208) |
| VV to VP | 0.0054 | (-0.0050-0.0158) | 0.0025 | (-0.0262-0.0311) |
| VV to PA | 0.0054 | (-0.0050-0.0158) | 0.0097 | (-0.0454-0.0648) |
| VV to CO | 0.0054 | (-0.0050-0.0158) | 0.0065 | (-0.0243-0.0372) |
| VV to MA | 0.0054 | (-0.0050-0.0158) | 0.0110 | (-0.0690-0.0911) |
| VV to MU | 0.0054 | (-0.0050-0.0158) | 0.0254 | (-0.0561-0.1070) |
| VV to CA | 0.0055 | (-0.0051-0.0161) | 0.0096 | (-0.0432-0.0625) |
| CA to GT | 0.0068 | (-0.0063-0.0199) | 0.0004 | (-0.0003-0.0010) |
| CA to CC | 0.0068 | (-0.0061-0.0197) | 0.0135 | (-0.0217-0.0488) |
| CA to EC | 0.0068 | (-0.0065-0.0201) | 0.0008 | (-0.0124-0.0141) |
| CA to AD | 0.0068 | (-0.0063-0.0199) | 0.0043 | (-0.0258-0.0344) |
| CA to VP | 0.0068 | (-0.0061-0.0197) | 0.0123 | (-0.0078-0.0324) |
| CA to PA | 0.0069 | (-0.0064-0.0202) | 0.0018* | (0.0006-0.0031) |
| CA to CO | 0.0068 | (-0.0061-0.0197) | 0.0009* | (0.000-0.0018) |
| CA to MA | 0.0068 | (-0.0063-0.0199) | 0.0001 | (-0.004-0.0046) |
| CA to MU | 0.0067 | (-0.0062-0.0196) | 0.0017 | (-0.0028-0.0062) |
| CA to VV | 0.0067 | (-0.0062-0.0196) | 0.0084 | (-0.0319-0.0488) |
| Mean | 0.0058* | (0.0046-0.0071) | 0.0053 | (-0.0039-0.0146) |
